# Effectiveness of radiotherapy after breast-conserving surgery in older patients with T1-2N0 breast cancer

**DOI:** 10.1007/s10549-019-05412-8

**Published:** 2019-08-26

**Authors:** Anna Z. de Boer, Esther Bastiaannet, Nienke A. de Glas, Perla J. Marang-van de Mheen, Olaf M. Dekkers, Sabine Siesling, Linda de Munck, Kelly M. de Ligt, Johanneke E. A. Portielje, Gerrit Jan Liefers

**Affiliations:** 1 Department of Surgery, Leiden University Medical Center, Location J10-71, Postzone K6-R, P.O. Box 9600, 2300 RC Leiden, The Netherlands; 2 Department of Medical Oncology, Leiden University Medical Center, Leiden, The Netherlands; 3 Department of Medical Decision-Making, Leiden University Medical Center, Leiden, The Netherlands; 4 Department of Clinical Epidemiology, Leiden University Medical Center, Leiden, The Netherlands; 5 Department of Research and Development, Netherlands Comprehensive Cancer Organisation, Utrecht, The Netherlands; 6 Department of Health Technology and Services Research, Technical Medical Center, University of Twente, Enschede, The Netherlands

**Keywords:** Breast cancer, Older patients, Breast-conserving treatment, Locoregional recurrence

## Abstract

**Purpose:**

In the Netherlands, radiotherapy after breast-conserving surgery (BCS) is omitted in up to 30% of patients aged ≥ 75 years. Although omission of radiotherapy is considered an option for older women treated with endocrine treatment, the majority of these patients do not receive systemic treatment following Dutch treatment guidelines. Therefore, the aim of this study was to evaluate the effect of omission of radiotherapy on locoregional recurrence risk in this patient population.

**Methods:**

Patients aged ≥ 75 years undergone BCS for T1-2N0 breast cancer diagnosed between 2003 and 2009 were selected from the Netherlands Cancer Registry. To minimize confounding by indication, hospital variation was used to assess the impact of radiotherapy-use on locoregional recurrence risk using cox proportional hazards regression. Hazards ratios with 95% confidence interval (CI) were estimated.

**Results:**

Overall, 2390 patients were included. Of the patients with hormone receptor-positive breast cancer, 39.3% received endocrine treatment. Five-year incidences of locoregional recurrence were 1.9%, 2.8%, and 3.0% in patients treated at hospitals with higher (average radiotherapy-use 96.0%), moderate (88.0%), and lower radiotherapy-use (72.2%) respectively, and nine-year incidences were 2.2%, 3.1%, and 3.2% respectively. Adjusted hazard ratios were 1.46 (95% CI 0.77–2.78) and 1.50 (95% CI 0.79–2.85) for patients treated at hospitals with moderate and lower radiotherapy-use, compared to patient treated at hospitals with higher radiotherapy-use.

**Conclusions:**

Despite endocrine treatment in only 39.3%, locoregional recurrence risk was low, even in patients treated at hospitals with lower radiotherapy-use. This provides reasonable grounds to consider omission of radiotherapy in patients aged ≥ 75 years with T1-2N0 breast cancer.

**Electronic supplementary material:**

The online version of this article (10.1007/s10549-019-05412-8) contains supplementary material, which is available to authorized users.

## Introduction

Breast-conserving surgery (BCS) followed by radiotherapy is the standard treatment for early stage breast cancer. However, various randomized clinical trials (RCTs) have investigated omission of the radiotherapy in older patients as the additional benefit is expected to decrease with declining residual life expectancy and increasing risk of dying from other causes with age [1–3]. These RCTs demonstrated a small benefit in locoregional control from radiotherapy, but no effect on distant metastasis-free or disease-specific survival.

As no survival benefit was demonstrated and locoregional recurrences can be treated with surgery, in 2004, omission of radiotherapy was incorporated in the National Comprehensive Cancer Network (NCCN) guideline as treatment option for patients aged ≥ 70 years with stage 1 breast cancer provided that they are treated with endocrine therapy [4]. However, this recommendation had only limited effect on radiotherapy-use in clinical practice [5]. Furthermore, other guidelines such as recommendations from the Society of Geriatric Oncology (SIOG) and European Society of Breast Cancer Specialists (EUSOMA) still state that radiotherapy should be considered in all elderly patients because it decreases the risk of locoregional recurrence [6].

The reluctance regarding omission of radiotherapy could be partially explained by concerns of clinicians about lower endocrine therapy-use and adherence in the true older population of patients with breast cancer compared to trial populations [7, 8]. The RCTs exclusively included patients using endocrine therapy [1, 2]. Moreover, adherence to endocrine treatment was supposedly higher than in the general older population. Although the aim of endocrine therapy is to reduce the risk of distant metastasis and improve breast cancer specific survival, the systemic therapy may also have a locoregional effect.

In the Netherlands, radiotherapy after BCS is omitted in up to 30% of patients aged ≥ 75 years, and the majority of these patients do not receive systemic treatment following Dutch treatment guidelines [9]. On the one hand, the omission of radiotherapy in the absence of endocrine treatment may potentially result in higher locoregional recurrence risks. On the other hand, older patients participating in trials are often a relatively young and healthy selection of the general older population [10]. Due to higher competing mortality risks in the general older population, the radiotherapy benefit may actually be smaller than demonstrated in the selected trial populations.

Population-based data can give important insight in the effectiveness of radiotherapy after BCS for the general older population, provided that confounding by indication is appropriately handled. Because confounding by unmeasured factors was expected, a method which can avoid such confounding was considered most effective in obtaining a valid effect estimate. Therefore, the aim of this study was to assess the effect of omission of radiotherapy after BCS on locoregional recurrence risk in patients aged ≥ 75 years with T1-2N0 breast cancer using hospital variation in radiotherapy-use as an instrumental variable-like approach.

## Methods

All patients aged ≥ 75 years who underwent BCS for T1-2N0 breast cancer between 2003 and 2009 were selected from the Netherlands Cancer Registry (NCR) and included in this study. The NCR is a database on cancer diagnosis and treatment. It is hosted by the Netherlands Comprehensive Cancer Organisation (IKNL) and receives reports of diagnosed malignancies from the nationwide network and registry of histopathology and cytopathology in the Netherlands (PALGA), which are confirmed and completed by the national hospital discharge databank. Trained data managers of the IKNL regularly collect data on diagnosis, staging, and treatment from medical records using international coding rules. In addition, information on recurrence status and comorbidity is collected for specific research purposes.

Breast cancer stage is defined according to the TNM Classification of Malignant Tumors for breast cancer (6th edition) [11]. Clinical T or N stage is used if pathological T or N stage is unknown. Recurrences are defined according to consensus-based definitions for classification of breast cancer recurrence [12]. Ipsilateral breast, chest wall, axillary, and supraclavicular lymph nodes recurrence are considered a locoregional recurrence. For the current study, recurrence status was available for a minimum of five years after diagnosis for all patients. We used a Landmark approach to avoid bias due to immortal time between diagnosis, surgery and radiotherapy. Therefore, follow-up started 3 months after diagnosis. Endpoint for follow-up was time of recurrence, death, or last follow-up visit, whichever came first. Vital status was available until January 31st 2017 through linkage of NCR data with the Municipal Personal Records database. Information on comorbidity at time of diagnosis was retrospectively collected for patients diagnosed during incidence years 2007–2009.

### Hospital radiotherapy variation

We used an instrumental variable-like approach to minimize confounding by indication by using hospital variation in radiotherapy-use [13]. Treatment decisions in older patients with breast cancer are influenced by aspects of general health such as physical and cognitive functioning, which also affect outcome. As information regarding these factors is not available in cancer registries, conventional statistical methods are unable to take these factors into account. Consequently, results are at high risk of bias due to residual confounding [13]. To minimize this problem, we used variation in radiotherapy-use among hospitals in which patients underwent surgery to assess the effect of radiotherapy. We assumed that hospitals are independent of breast cancer related prognostic factors, given that all hospitals in the Netherlands provide breast cancer care and older patients generally go to the nearest hospital. Three groups were constructed using tertiles of radiotherapy-use, based on the percentage of patients treated with radiotherapy within each hospital: higher level (range 92.3–100%), moderate (range 83.3–92.3%), and lower (range 0–83.3%) radiotherapy-use hospitals. Characteristics of patients treated at higher, moderate, and lower radiotherapy-use hospitals were presented. The characteristics of patients who were treated with and without radiotherapy were also presented to demonstrate the effect on confounding of using hospital variation instead of comparing treated and untreated patients directly.

### Statistical analysis

Statistical analysis was performed with SPSS 23.0 and STATA 12.1. Multiple imputation by chained equation was performed to account for missing values, assuming that data were missing at random [14]. Imputation models were applied including incomplete and complete variables. Analyses were based on the pooled results of 25 imputed sets according to Rubin’s rules [15]. Pearson’s *χ*^2^ tests were used to assess differences in characteristics between patients who were treated with and without radiotherapy, and between patients treated at hospitals with different levels of radiotherapy-use. Cumulative incidences of locoregional recurrence were calculated using the Cumulative Incidence Competing Risk method, considering distant recurrence and death without recurrence as competing events [16]. Cox proportional hazards models were used to estimate hazard ratios (HRs) with 95% confidence intervals (CIs) to compare locoregional recurrence risk in patients treated at hospitals with different levels of radiotherapy-use. The higher radiotherapy-use group was used as reference group. We adjusted by multivariable analysis for imbalances that were statistically significant. The scaled Schoenfeld residuals of the covariates over time were tested for a non-zero slope in a generalized linear regression. No violations were found. As recurrence status for patients diagnosed between 2003 and 2006 was not available after 5 years, a sensitivity analysis was performed with follow-up truncated at 5 years. To avoid immortal time bias, a Landmark approach was used, starting follow-up at 3 months after diagnosis. All statistical tests were two-sided.

## Results

Overall, 2390 patients with T1-2N0 breast cancer aged ≥ 75 years were included. Median age was 79.2 years [interquartile range (IQR) 76.4–82.5 years]. Table 1 shows clear differences in characteristics between patients treated with and without radiotherapy. Patients treated with radiotherapy were younger and had less comorbidity compared to patients treated without radiotherapy. With regard to tumor characteristics, patient treated with radiotherapy had smaller tumors, more often hormone receptor-positive tumors, and surgery was irradical in fewer patients. Furthermore, only 32.6% of the patients treated with radiotherapy received endocrine therapy, compared to 54.7% in patient treated without radiotherapy (*p *= 0.023). Notably, of the patients with hormone receptor-positive tumors in this study, 39.3% received endocrine treatment.

**Table 1:** Characteristics of patients treated with and without radiotherapy

	Radiotherapy		No radiotherapy		*p* value
	*n* = 2039		*n* = 351		
	*N* (%)	(%)^a^	*N* (%)	(%)^a^	
Age at diagnosis					**< 0.001**
75–79	1286 (63.1)		69 (19.7)		
80–84	627 (30.8)		109 (31.1)		
> 85	126 (6.2)		173 (49.3)		
CCI					**0.001**
0	531 (26.0)	(58.3)	52 (14.8)	(38.6)	
1	192 (9.4)	(24.0)	39 (11.1)	(35.6)	
> 2	133 (6.6)	(17.7)	30 (8.6)	(25.8)	
Unknown	1183 (58.0)		230 (65.5)		
Tumor grade					0.455
1	570 (28.0)	(30.4)	100 (28.5)	(32.2)	
2	929 (45.6)	(48.8)	132 (37.6)	(42.0)	
3	407 (20.0)	(20.8)	85 (24.2)	(25.8)	
Unknown	133 (6.5)		34 (9.7)		
T stage					**< 0.001**
T1	1449 (71.1)		213 (60.7)		
T2	590 (28.9)		138 (39.3)		
HR expression					**0.036**
ER + and/or PR+	1682 (82.5)	(88.9)	280 (79.8)	(84.9)	
ER− and PR−	207 (10.2)	(11.1)	48 (13.7)	(15.1)	
Unknown	150 (7.4)		23 (6.6)		
Her2Neu overexpression					0.435
Negative	1283 (62.9)	(91.5)	208 (59.3)	(89.6)	
Positive	106 (5.2)	(8.5)	19 (5.4)	(10.4)	
Unknown	650 (31.9)		124 (35.3)		
Surgical margins					**< 0.001**
Free	1912 (93.8)		302 (86.0)		
Not free	91 (4.5)		33 (9.4)		
Unknown	36 (1.8)		16 (4.6)		
Adjuvant endocrine therapy in HR+					**0.023**
Yes	565 (33.6)	(32.6)	157 (56.1)	(54.7)	
No	1117 (66.4)	(67.4)	123 (43.9)	(45.3)	
Chemotherapy					0.560
Yes	3 (0.2)		1 (0.3)		
No	2036 (99.9)		350 (99.7)		
Type of hospital					0.066
University hospital	146 (7.2)		35 (10.0)		
Non-university hospital	1892 (92.8)		316 (90.0)		

Bold values represent significant *p*-values

*CCI* Charlson Comorbidity Index, *HR* hormone receptor

^a^Proportional distribution after multiple imputation

The patients were divided into tertiles based on radiotherapy-use within each hospital (Table 2). The average radiotherapy-use was 96.0% in the higher-use, 88% in the moderate-use, and 72.2% in the lower-use hospitals. The groups included patients from 46, 35, and 47 different hospitals respectively. Comorbidity, an important determinant of receiving radiotherapy, and tumor characteristics were equally distributed over the groups. An imbalance in age distribution remained, patients treated in lower-use hospitals were older (17.8% of the patients was aged > 85 years) compared to patients treated in higher-use and moderate-use hospitals (8.4% and 11.2%, *p *< 0.001). Furthermore, endocrine treatment was more often prescribed in patients treated in lower-use hospitals (40.0%) compared to patients treated in higher-use and moderate-use hospitals (34.3% and 32.5% respectively, *p *= 0.023). Another imbalance was observed for type of hospital as academic hospitals were overrepresented in the lower-use group (14.2% compared to 4.6% in the higher-use and 3.7% in the moderate-use group, *p* < 0.001).

**Table 2:** Characteristics of patients by tertile of hospital radiotherapy-use

	Higher-use		Moderate-use		Lower-use		*p* value
	*n* = 802		*n* = 775		*n* = 813		
	*n* (%)	(%)^a^	*n* (%)	(%)^a^	*n* (%)	(%)^a^	
Radiotherapy	770 (96.0)		682 (88.0)		587 (72.2)		**< 0.001**
Age at diagnosis							**< 0.001**
75–79	479 (59.7)		449 (57.9)		427 (52.5)		
80–84	256 (31.9)		239 (30.8)		241 (29.6)		
> 85	67 (8.4)		87 (11.2)		145 (17.8)		
CCI							0.154
0	230 (28.7)	(57.9)	188 (24.3)	(56.4)	165 (20.3)	(52.0)	
1	78 (9.7)	(23.2)	73 (9.4)	(25.4)	80 (9.8)	(28.6)	
> 2	66 (8.2)	(18.9)	46 (5.9)	(18.3)	51 (6.3)	(19.4)	
Unknown	428 (53.4)		468 (60.4)		517 (63.6)		
Tumor grade							0.083
1	243 (30.3)	(32.5)	224 (28.9)	(31.9)	203 (25.0)	(27.7)	
2	353 (44.0)	(47.1)	327 (42.2)	(45.9)	381 (46.9)	(50.4)	
3	155 (19.33)	(20.4)	166 (21.4)	(22.3)	171 (21.0)	(21.9)	
Unknown	51 (6.4)		58 (7.5)		58 (7.1)		
T stage							0.822
T1	564 (70.3)		534 (68.9)		564 (69.4)		
T2	238 (29.7)		241 (31.1)		249 (30.6)		
HR expression							0.699
ER + and/or PR+	674 (84.0)	(89.8)	612 (79.0)	(86.0)	676 (83.2)	(89.2)	
ER− and PR−	77 (9.6)	(10.2)	97 (12.5)	(14.0)	81 (10.0)	(10.9)	
Unknown	51 (6.4)		66 (8.5)		56 (6.9)		
Her2Neu overexpression							0.692
Negative	519 (64.7)	(92.2)	478 (61.7)	(90.0)	494 (60.8)	(91.5)	
Positive	39 (4.9)	(7.9)	47 (6.1)	(10.0)	39 (4.8)	(8.5)	
Unknown	244 (30.4)		250 (32.3)		280 (34.44)		
Surgical margins							0.465
Free	747 (93.1)		723 (93.3)		744 (91.5)		
Not free	42 (5.2)		35 (4.5)		47 (5.8)		
Unknown	13 (1.6)		17 (2.2)		22 (2.7)		
Adjuvant endocrine therapy in HR+							**0.023**
Yes	238 (35.3)	(34.3)	202 (33.0)	(32.5)	282 (41.7)	(40.0)	
No	436 (64.7)	(65.7)	410 (67.0)	(67.5)	394 (58.3)	(60.0)	
Chemotherapy							0.186
Yes	1 (0.1)		0 (0)		3 (0.4)		
No	801 (99.9)		775 (100)		810 (99.6)		
Type of hospital							**< 0.001**
University hospital	37 (4.6)		29 (3.7)		115 (14.2)		
Non-university hospital	764 (95.4)		746 (96.3)		698 (85.9)		

Bold values represent significant *p*-values

*CCI* Charlson Comorbidity Index, *HR* hormone receptor

^a^Proportional distribution after multiple imputation

**Fig. 1:**
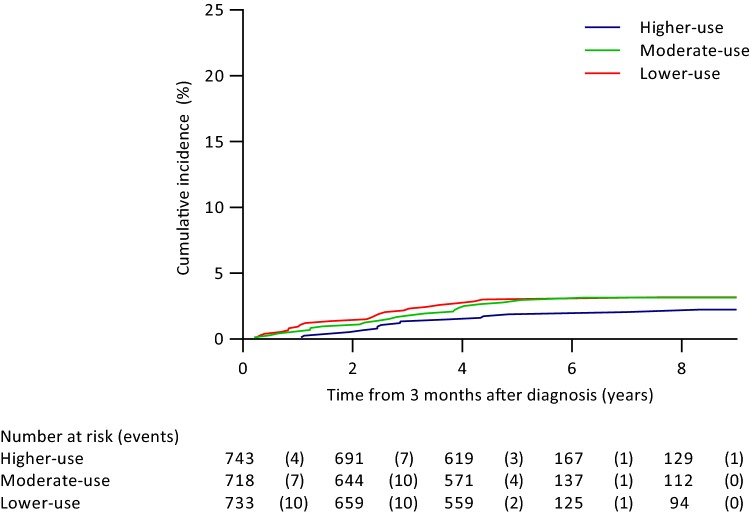
Cumulative incidence of locoregional recurrence in high-use, moderate-use, and low-use radiotherapy hospitals

Out of the 2390 patients, 186 patients were lost to follow-up and 10 patients died during the first 3 months after diagnosis. For the 2194 patients included in the time-to-event analysis (Landmark approach), median follow-up was 4.8 years starting from 3 months after diagnosis (IQR 4.8–4.8, range 0.03–10.8 years), during which 61 patients had a locoregional recurrence. Cumulative incidences of locoregional recurrence by hospital level radiotherapy-use are graphically represented in Fig. 1. Five-year cumulative incidences were 1.9%, 2.8%, and 3.0% in the higher-use, moderate-use and lower-use group, and nine-year cumulative incidences were 2.2%, 3.1%, and 3.2% respectively (Table 3).

**Table 3:** Cox proportional hazards analysis for time to locoregional recurrence by hospital radiotherapy-use

	Cumulative incidences (95% CI)			
	Five-year follow-up^a^	Nine-year follow-up^a^	Univariable HR^b^ (95% CI)	Multivariable HR^b,c^ (95% CI)
Higher-use	1.9 (1.1–3.1)	2.2 (1.3–3.6)	Reference	Reference
Moderate-use	2.8 (1.8–4.2)	3.1 (2.0–4.6))	1.49 (0.78–2.83)	1.46 (0.77–2.78)
Lower-use	3.0 (1.9–4.4)	3.2 (2.1–4.7)	1.55 (0.82–2.94)	1.50 (0.79–2.85)

*HR* hazard ratio, *CI* confidence interval

^a^Follow-up from landmark at 3 months after diagnosis

^b^Calculated with complete follow-up time

^c^Adjusted for age (continuous), endocrine therapy and type of hospital

Results of the Cox proportional hazards analysis are shown in Table 3. In univariable analysis, the HRs were 1.49 (95% CI 0.78–2.83) and 1.55 (95% CI 0.82–2.94) for patients treated at hospitals with moderate and lower radiotherapy-use respectively, compared to patients treated at hospitals with higher radiotherapy-use. After adjustment for age, endocrine treatment, and type of hospital, the HRs were 1.46 (95% CI 0.77–2.78) and 1.50 (95% CI 0.79–2.85) respectively. The sensitivity analysis with truncated five-year follow-up demonstrated comparable HRs compared to the primary adjusted analysis: HR 1.50 (95% CI 0.76–2.96) and 1.59 (95% CI 0.81–3.14) (Supplementary Table).

## Discussion

The present study shows that locoregional recurrence rates are low in patients aged ≥ 75 years who underwent BCS, even in patients treated in hospitals with lower radiotherapy-use. No association was found between radiotherapy-use and locoregional recurrence risk.

Our study adds to available evidence, since the low locoregional recurrence risks that were seen in previous RCTs were confirmed in this population-based cohort in which only 39.3% of the patients was treated with endocrine therapy. Therefore, concerns of an increased locoregional recurrence risk among older patients not treated with endocrine therapy are contradicted. We argue that this can be explained by the declining residual life expectancy and increasing risk of dying from other causes than breast cancer, so-called competing mortality, among the older population of patients with breast cancer [17].

The low locoregional recurrence rates reported in this study support the allowance of omission of radiotherapy in patients aged ≥ 75 years, even when patients are not treated with endocrine treatment. This is strengthened by the fact that we found locoregional recurrence risks in patients treated in hospitals with higher radiotherapy-use (average 96%) in our study (1.9% after 5 and 2.2% after 9 years), that were similar to patients in the radiotherapy-arm of the CALGB 9343 trial (1% after 5 and 2% after 10 years). This hallmark trial randomized patients aged ≥ 70 years with T1N0 breast cancer using endocrine treatment between radiotherapy or no radiotherapy after BCS. The trial exclusively included patients receiving endocrine treatment, whereas only 39.3% of the patients in our study was not treated with endocrine treatment conform Dutch treatment guidelines. Moreover, adherence to endocrine treatment was likely more typical for the true older population as population-based data were used.

Although RCTs provide the highest level of evidence for treatment efficacy, their external validity is often questioned. Therefore, results from observational studies can add to the generalizability. However, all observational studies are susceptible for confounding by indication because treatment allocation is likely based on reasons associated with outcomes. The validity of the results strongly depends on the ability to reduce such confounding.

Especially in older populations, directly comparing patients who are treated differently leads to biased effect estimates as treatment decisions are made on the combination and interaction of disease and patient related factors for which it appears impossible to adjust [13]. Furthermore, information on important confounding factors may be missing in observational studies, while conventional methods to reduce confounding such as multivariable analysis or propensity score matching rely on measured variables. Consequently, aspects of general health such as comorbidity, physical and cognitive functioning are often not taken into account. As a result, using conventional methods generally results in an overestimation of effect estimates, and may even demonstrate an opposite causal effect [13, 18, 19].

Many previous observational studies addressed the omission of radiotherapy after BCS in older patients. Some advocate that radiotherapy may be omitted [20–23], whereas others state that it is unsafe due to a higher risk of locoregional recurrence [24–26] or even worse breast cancer specific and overall survival [25, 27–29]. Although different patient selections could play a role in the varying findings, results of these studies using conventional methods to adjust for confounding may have been biased to some extent. For example, the worse overall survival in patients treated without radiotherapy (not found in RCTs) could be in fact a reflection of the lower probability to receive radiotherapy in patients with higher competing mortality risk [25, 28]. Furthermore, even when disease-specific outcomes are used, confounding by indication can still cause bias through differential censoring of patients dying from other causes [30].

Instead of a conventional statistical approach, we used an instrumental variable-like approach by using hospital variation in radiotherapy-use to minimize confounding by indication [13]. We demonstrated that patients treated with and without radiotherapy differed in many aspects, but using hospital variation, the constructed radiotherapy groups were fairly similar. Comorbidity is an important confounding factor as it strongly influences whether a patient receives treatment, and at the same time, affects survival and disease-specific outcomes such as locoregional recurrence risk indirectly. Therefore, the fact that the groups were similar concerning comorbidity indicates that confounding by comorbidity was effectively resolved. Notably, we expected patients not treated with radiotherapy to have more favorable tumor characteristics, but on the contrary, we observed larger tumors and less hormone receptor-positivity. This may imply that the decision for radiotherapy depends more on patient related factors than on tumor characteristics.

Our study has important limitations. Foremost, although using hospital variation may result in more valid results, we could only assess the effect of a difference of 23.8% in radiotherapy-use. Consequently, the results apply to patients in whom the decision for radiotherapy was influenced by hospital variation, but this selection is not readily identifiable. However, we do not advocate that radiotherapy should be omitted in all patients, but rather advise against routinely treating all older patients with radiotherapy. Second, the low event rate prevented us from exploring subgroups with a differential radiotherapy-use effect. Third, residual confounding could not be completely ruled out because some imbalances between the radiotherapy-use groups persisted. For this reason, we also performed a multivariable analysis. Last, the absolute risk of locoregional recurrence for patients treated without radiotherapy could not be provided as a proportion of the patients treated in the lower-use hospitals still received radiotherapy.

To obtain the absolute locoregional recurrence risk for patients in whom radiotherapy after BCS is omitted, the ongoing TOP-1 (Tailored treatment in Older Patients) study (BOOG study number 2016-01) was recently initiated and is currently running in almost all breast cancer clinics in the Netherlands. This prospective cohort study includes patients aged ≥ 70 years with endocrine receptor-positive grade 1 tumors up to 2 cm and grade 2 tumors up to 1 cm who are treated without radiotherapy after BCS, and assesses whether the LRR remains below the prespecified limit of 3.9%. Notably, none of these patients is treated with endocrine therapy following Dutch treatment guidelines. To be able to assess the generalizability of the results, all patients are characterized by a geriatric assessment. Secondary outcomes are quality of life and toxicity.

In conclusion, despite endocrine treatment being prescribed in only 39.3% of the patients, locoregional recurrence risk after BCS in patients aged ≥ 75 years with T1-2N0 breast cancer was low, even in patients treated at hospitals with lower radiotherapy-use. Our study provides reasonable grounds to consider omission of radiotherapy after BCS. At older age, the frequent hospital visits required for radiotherapy can prove a substantial burden due to impaired mobility, lack of transportation, lack of social support, and caregiver responsibilities. Therefore, instead of routinely admitting radiotherapy after BCS, a shared-decision making approach is appropriate in all patients aged ≥ 75 years with T1-2N0 breast cancer.

## Electronic supplementary material

Below is the link to the electronic supplementary material.

Supplementary material 1 (DOCX 13 kb)
