# Supplementary material for: Effectiveness of radiotherapy after breast-conserving surgery in older patients with T1-2N0 breast cancer
Source: Breast Cancer Res Treat. 2019 Aug 26;178(3):637–45. doi: 10.1007/s10549-019-05412-8 (PMC6817758; doi:10.1007/s10549-019-05412-8)
Supplement: Supplementary file 1 — Supplementary material 1 (DOCX 13 kb) [file 10549_2019_5412_MOESM1_ESM.docx]

| **Supplementary Table.** Sensitivity analysis. Cox proportional hazards analysis for time to locoregional recurrence by hospital radiotherapy-use with truncated five year follow-up. | | |
| --- | --- | --- |
|  | Univariable HR (95% CI) | Multivariable HR^a^ (95% CI) |
| Higher-use | Reference | Reference |
| Moderate-use | 1.53 (0.77-3.03) | 1.50 (0.76-2.96) |
| Lower-use | 1.67 (0.85-3.26) | 1.59 (0.81-3.14) |
| ^a^Adjusted for age (continuous), endocrine therapy and type of hospital. HR: hazard ratio, CI: confidence interval. | | |

*Effectiveness of radiotherapy after breast-conserving surgery in older patients with T1-2N0 breast cancer*, Breast Cancer Research and Treatment, Anna Z. de Boer, Esther Bastiaannet, Nienke A. de Glas, Perla J. Marang-van de Mheen, Olaf M. Dekkers, Sabine Siesling, Linda de Munck, Kelly M de Ligt, Johanneke E.A. Portielje, Gerrit Jan Liefers, Department of Surgery, Leiden University Medical Center, Leiden, The Netherlands, a.z.de_boer@lumc.nl
